# Recent Kinematic and Kinetic Advances in Olympic Alpine Skiing: Pyeongchang and Beyond

**DOI:** 10.3389/fphys.2019.00111

**Published:** 2019-02-20

**Authors:** Matej Supej, H.-C. Holmberg

**Affiliations:** ^1^Faculty of Sport, University of Ljubljana, Ljubljana, Slovenia; ^2^The Swedish Winter Sports Research Centre, Mid Sweden University, Östersund, Sweden; ^3^School of Sport Sciences, UiT The Arctic University of Norway, Tromsø, Norway

**Keywords:** downhill, giant slalom, performance, super-G, tactics, technique

## Abstract

Alpine skiing has been an Olympic event since the first Winter Games in 1936. Nowadays, skiers compete in four main events: slalom, giant slalom, super-G and downhill. Here, we present an update on the biomechanics of alpine ski racers and their equipment. The technical and tactical ability of today’s world-class skiers have adapted substantially to changes in equipment, snow conditions and courses. The wide variety of terrain, slopes, gate setups and snow conditions involved in alpine skiing requires skiers to continuously adapt, alternating between the carving and skidding turning techniques. The technical complexity places a premium on minimizing energy dissipation, employing strategies and ski equipment that minimize ski-snow friction and aerodynamic drag. Access to multiple split times along the racing course, in combination with analysis of the trajectory and speed provide information that can be utilized to enhance performance. Peak ground reaction forces, which can be as high as five times body weight, serve as a measure of the external load on the skier and equipment. Although the biomechanics of alpine skiing have significantly improved, several questions concerning optimization of skiers’ performance remain to be investigated. Recent advances in sensor technology that allow kinematics and kinetics to be monitored can provide detailed information about the biomechanical factors related to success in competitions. Moreover, collection of data during training and actual competitions will enhance the quality of guidelines for training future Olympic champions. At the same time, the need to individualize training and skiing equipment for each unique skier will motivate innovative scientific research for years to come.

## Introduction

Alpine skiing, a physically, technically and tactically complex and challenging sport, has been an Olympic event since the first Winter Games in Garmisch-Partenkirchen, Germany, in 1936. More effective training and advances in equipment and snow preparation have improved the performance of Olympic alpine skiers dramatically since then. Winning margins are now often no more than fractions of a second and biomechanical factors determine which skiers win medals.

This sport involves the technical events slalom (SL) and giant slalom (GS) and speed events super giant slalom (SG) and downhill (DH), each with its own gate placement (and thereby turning radii), terrain, speed, and course length, some of which are regulated by the International Ski Federation (FIS) (Gilgien et al., 2015; Supej et al., 2015; Erdmann et al., 2017). In the case of SL the speed is 40–60 km/h, whereas the maximal speeds in GS, SG and DH average 70 (80), 80 (102), and 86 (120) km/h, respectively (Gilgien et al., 2015). Typical race durations are approximately 2 × 50–60 s for the SL, 2 × 70–90 s for the GS, 1 × 80 s for the Super-G, 1 × 120 s for the DH, 1 × 40–45 s (SL) and 1 × 80–120 s (DH) for the combined event and 4 × 20 s for the team parallel slalom ^1^. Official data from the Pyeongchang Olympic Games 2018 are presented in Table 1.

**Table 1:** Characteristics of the alpine ski racing events at the Pyeongchang Olympic Games in 2018.

	Course		Vertical		Average		Best run time		Number of gates	
Event	length (m)		drop (m)		gradient (°)		(min:sec:hundredths)		(1st or 1st/2nd run)	
	M	W	M	W	M	W	M	W	M	W
Slalom	575	556	211	204	36.7	36.7	1:38:99	1:38:63	66/66	63/63
Giant slalom	1326	1250	440	400	33.2	32	2:18:04	2:20:02	53/53	51/51
Super-G	2322	2010	650	585	28	29.1	1:24:44	1:21:11	45	43
Combined										
Slalom	521	515	200	179	38.4	34.8	45.96	40:23	60	N/A
Downhill	2050	2775	650	730	31.7	26.3	1:19.24	1:40:11	25	38
Downhill	2965	2775	825	730	27.8	26.3	1:40:25	1:39:22	33	38
Team event	265	265	80	80	30.2	30.2	N/A	N/A	26	26


*M, men; W, women; N/A, not available*.

To achieve the shortest combined time on all sections of a course and thereby win, the alpine skier should (1) lose as little time as possible on his/her weakest sections and win as much as possible on strong sections or (2) approach the best time on all sections (Supej and Cernigoj, 2006; Hébert-Losier et al., 2014).

**FIGURE 1:**
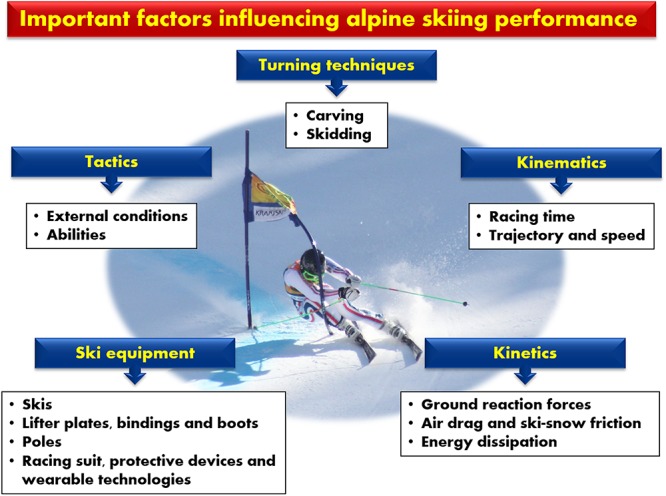
Biomechanical determinants of the performance of Olympic alpine skiers.

The technical complexity involved in continuously adapting turning technique to changes in terrain, slope, gate setup, and snow conditions demands biomechanical analysis of the determinants of elite performance that is more detailed and nuanced than that based on racing time alone (Supej, 2008; Supej et al., 2011; Federolf, 2012; Spörri et al., 2018). This is challenging, since many kinematic and kinetic factors influence performance directly or indirectly (Figure 1), including the trajectory of the skis and/or center of mass, turning radius and speed, ground reaction forces (GRF), aerodynamic drag and frictional forces, as well as energy dissipation (i.e., the efficiency of mechanical energy utilization) (Supej et al., 2005, 2011, 2013, 2015; Supej, 2008; Supej and Holmberg, 2010; Federolf, 2012; Meyer et al., 2012; Hébert-Losier et al., 2014; Spörri et al., 2018). In addition, biomechanical differences between the various turning techniques, the inter-dependency of turns, tactics and ski equipment are important considerations in this context (Supej et al., 2002, 2004; Supej and Cernigoj, 2006; Chardonnens et al., 2010).

Our aim here was to provide an update on the biomechanics of alpine ski racers and the equipment they use.

## Turning Techniques

Prior to the Winter Olympics in Nagano in 1998, alpine skiers utilized so-called classic skis with a side-cut radius longer than approximately 30 m. For many years short turns around gates with straight skiing between turns was considered optimal for SL and GS. However, already in the 1980s, skiers began striving for so-called clean turns (now known as carving turns). For example, when the movements of Alberto Tomba (the dominant skier in technical events during the late 1980s and 1990s, with gold medals in slalom and giant slalom at the World Championships and Olympic Games) were analyzed on the basis of slow-motion video recordings and images, coaches realized that he placed more pressure on the tails of the skis after the fall line, enabling “carving” (i.e., cutting into the snow, so that the skis bend into an arc and then turn). The translocation of pressure from the forefoot (at the beginning of the turn) toward the heel (at the end of the turn) is still a feature of alpine ski racing (Falda-Buscaiot et al., 2017).

Since the introduction of carving skis, this type of turn was developed further, resulting in novel features such as the “single motion” technique in slalom (Supej et al., 2002, 2004; Müller and Schwameder, 2003) and “cross-under” technique in giant slalom (Chardonnens et al., 2010). With both of these techniques, the posture of the skier’s body while transferring weight is more “crunched” than when rounding the gate, which is the opposite of the situation with earlier elite skiers. More specifically, with the “single motion” technique, the skier starts to extend his/her body after the transfer of weight and continues this extension during the early steering phase; flexion of the body begins soon after the fall line; and, finally, the skier is most “crunched” up during the subsequent transfer of weight (Supej et al., 2002, 2004). Such “harmonious” movement incorporates both a single extension and single flexion per each turn. With the “cross-under” technique used in giant slalom, the trunk remains stable during the transfer of weight, with movement of the legs altering the edges of the skis (Chardonnens et al., 2010). In contrast, with the techniques employed traditionally in giant slalom the trunk swings over the legs during the transfer of weight.

For two decades, in attempt to reduce injuries, the FIS has implemented new regulations concerning primarily the side-cut, length and waist width of skis, as well as the nature of the race course (Gilgien et al., 2015, 2016; Haaland et al., 2016; Kröll et al., 2016b,a; Spörri et al., 2016a,b, 2017; Supej et al., 2017). These regulations have influenced technique and tactics significantly, especially in the case of slalom and giant slalom. Consequently, in addition to smooth carving turns, today’s elite skiers utilize turns that involve skidding or so-called “free rotation” of the skis during the initiation and/or early steering phase.

## Kinematics

### Racing Time

In contrast to the single split time in the 1960s, today’s races involve 3–4 split times. Modern technology enables, e.g., gate-to-gate time analysis (Supej and Holmberg, 2011), revealing the gate or turn at which the skier loses or gains time. This type of analysis has demonstrated that a skier can lose as much as 0.4 s on the first few gates of a course and, moreover, that when a skier loses time on flat terrain, he/she can regain gate-to-gate times comparable to those of the fastest skier only many gates later. Similarly, the time required for elite skiers to navigate special gate combinations, such as close to and after hairpin bends in slalom, varies considerably.

Nevertheless, evaluating performance on the basis of racing time alone, even on short sections of a course, involves several limitations (Supej, 2008). This time is influenced by the skier’s initial velocity, position and orientation. Moreover, the position and orientation at the end of a section relative to the following gate, as well as the exit speed will exert little influence on section time, but may affect subsequent performance profoundly. Accordingly, other measures of performance are required.

### Trajectory and Speed

In general, skiing the shortest possible trajectory rapidly results in the fastest time (Supej, 2008; Federolf, 2012; Spörri et al., 2018). The ability to maintain high speed depends not only on the trajectory, but also on technique and tactics.

Usually, while often involving longer trajectories, faster and smoother turns are initiated higher up the slope and/or well before the gate, are completed closer to the gate and are longer (Brodie et al., 2008; Supej, 2008; Spörri et al., 2012b, 2018). Such turns generally allow greater acceleration out of/away from the gate and straighter subsequent skiing (Brodie et al., 2008), with faster entry into subsequent turns. Notably, instantaneous velocity is more influential than choice of trajectory or turning radius (i.e., the distance traveled) or, in other words, higher velocity is more advantageous than a shorter trajectory (Federolf, 2012).

## Kinetics

### Ground Reaction Forces

In alpine skiing, peak GRF, a common measure of the external load on the skier and equipment, can be as high as five times body weight in slalom (Supej et al., 2002). In the case of the other three major disciplines, the highest GRFs were observed during giant slalom, followed by super-G, with the lowest values during downhill racing (Gilgien et al., 2014). When turning, the GRFs are considerably higher during the steering than weight-transition phase, when they may even become zero if the skier loses ground contact (Supej et al., 2002, 2004; Reid, 2010; Vaverka et al., 2012; Falda-Buscaiot et al., 2017).

The distributions of GRFs for the best and less successful elite slalom skiers appear to be similar, although the most pronounced GRFs coincide with the lowest differential specific mechanical energy (i.e., highest energy dissipation/lower performance) (Supej et al., 2011). This is consistent with the observation that the shortest trajectory is not necessarily the fastest and may even be detrimental to the instantaneous performance of a skier, in particular during turns of short radius (Supej, 2008; Supej and Holmberg, 2010; Supej et al., 2011). Furthermore, slalom techniques involving both less zero GRF and lower maximal GRF are more efficient and faster (Supej et al., 2002; Hébert-Losier et al., 2014). These findings indicate that timing of GRFs may exert a pronounced impact on performance.

### Air Drag and Ski-Snow Friction

Aerodynamic drag and ski–snow friction are the only two mechanical forces that can have a detrimental impact on skiing performance (von Hertzen et al., 1997; Federolf et al., 2008; Meyer et al., 2012; Supej et al., 2013). Postures that minimize the exposed frontal area of a skier are key to reducing aerodynamic drag (Watanabe and Ohtsuki, 1977; Barelle et al., 2004), thereby elevating velocity (Watanabe and Ohtsuki, 1977) and reducing overall time (Watanabe and Ohtsuki, 1977; Luethi and Denoth, 1987). When skiing downhill, aerodynamic drag accounts for almost 50% of the differences in racing time between slower and faster skiers (Luethi and Denoth, 1987), whereas with giant slalom, this drag causes only 15% of the total energy loss per turn and is not considered a major determinant of performance (Supej et al., 2013). Aerodynamic drag becomes more important as the speed increases (e.g., from slalom to downhill) (Gilgien et al., 2013, 2018).

The opposite is true for ski-snow friction, which is more important at slower speeds, particularly when turning. During slalom and giant slalom races ski-snow friction dissipates most of the energy (Supej et al., 2013). Even in the speed disciplines, involving more intense turning, the skiers focus more on guiding the skis smoothly than minimizing the frontal area exposed.

### Energy Dissipation

Good turns are usually the result of effective usage of potential energy (i.e., minimization of ski-snow friction and aerodynamic drag in combination with optimizing ski trajectory). Such efficiency is particularly important in speed events and on the flat sections of most courses. However, in slalom and giant slalom, particularly on steeper slopes, minimization of energy dissipation does not necessarily ensure the shortest overall time. For elite skiers, minimization while maintaining high velocity and optimal trajectories on all sections also exerts a considerable impact on outcome.

Supej et al. (2008, 2011) reported that during a slalom event most energy is dissipated during steering in the vicinity of the gates and turns of short radius (< 15 m), and least during weight transition prior to initiation of a turn. In fact, during turns of short radius, the difference in specific mechanical energy is related directly to this radius (Supej et al., 2011), suggesting that longer turns may improve racing performance, as discussed above and consistent with the findings by Spörri et al. (2012b). Similarly, elite skiers optimize their use of potential energy more easily with carving than with skidding or pivoting turns (Supej, 2008).

To summarize, no individual biomechanical parameter can explain *why* one skier is faster than another (Hébert-Losier et al., 2014). Kinematic parameters reflect more the outcome of performance (i.e., without consideration of cause) and kinetic parameters the underlying causes. Elite skiers attempt to exploit these intricate interactions between biomechanical parameters and technique under varying conditions in a manner that minimizes descent times.

## Ski Equipment

### Skis

With respect to equipment, the continuous development of skis has influenced performance by elite alpine skiers most. For instance, when World Cup skiers first started to use “carving” skis in 1999, the smoother runs allowed faster skiing and shorter turns, particularly in slalom and giant slalom. In other disciplines, the length and side-cut radii increase with speed and turning radius (see Table 2). Moreover, enhanced awareness of injury and possible causes has led to regulation of the side-cut radii and waist width of skis by the FIS (Table 2) several times over the past decade (Burtscher et al., 2008; Spörri et al., 2012a, 2017; Haaland et al., 2016; Supej et al., 2017).

**Table 2:** International Ski Federation (FIS) regulations concerning the equipment and courses involved in international skiing competitions.

	EQUIPMENT								COURSE			
Event	Minimal ski length (cm)		Maximal ski waist width (cm)	Minimal side-cut radius (m)		Standing height (with ski/plate/binding) (mm)	Boot height (from sole to top of foot bed) (mm)	Distance between gates (m)	Vertical drop (m)		^∗^Number of gates
	M	W	M/W	M	W	M/W	M/W	M/W	M	W	M/W
Slalom	165	155	63^∗∗^	No rule	No rule	50	43	6–13^∗∗∗^	180–220	140–220	30–35%
Giant slalom	193	188	65	30	30	50	43	10–27	250–450	250–400	11–15%
Super-G	210	205	65	45	40	50	43	Minimally 25	400–650	400–600	Minimally 35
Downhill	218	210	65	50	50	50	43	No rule	800–1100	450–800	No rule

*M, men, W, women. ^∗^The number of gates in Slalom and Giant Slalom is 30% of the vertical drop (e.g., 30% of 200 m means 60 gates), while in Super-G only the minimal number of gates is specified. ^∗∗^The maximal ski waist is regulated in all the events except slalom, where the minimal width is regulated instead. ^∗∗∗^For special gate characteristics such as hairpins or delayed gates, the distances differ*.

Racing skis have predominately a sandwich construction with a wooden core. Today’s skis have a different overall geometry, contain more advanced materials and vary in camber curve. Thickness directly influences their longitudinal stiffness (Heinrich et al., 2011), which has been changing proportionally in response to side-cut radius regulations, particularly in GS. Improvements in construction and the servicing of metal edges of skis enable sharp and/or carving turns even on hard snow or ice (Brown, 2009). However, elite skiers have individual subjective preferences concerning longitudinal and torsional stiffness, as well as edge preparations.

### Lifter Plates, Bindings, and Boots

Lifter plates (between the ski and binding), introduced around the time of the Olympic Games in Calgary in 1988, allow more optimal bending. The associated increase in standing height allows more angling of the skis, in spite of regulation by the FIS (Table 2). Today’s plates also improve the torsional stiffness of skis, dampen vibrations and enhance the release of ski bindings (Senner et al., 2013; Supej and Senner, 2017).

Ski boots have also undergone important development. Newer plastics and molding enable thinner, more anatomic outer shells. In addition, boot-fittings have improved considerably, with individual liners and insoles, allowing better transfer of the skier’s action to the skis. The viscoelastic properties of ski boots, with moment-angle hysteresis, still cause energy dissipation (Eberle et al., 2016; Knye et al., 2016). It is more important that flexural stiffness be lower for downhill than technical disciplines, enabling better gliding and lower tuck.

### Poles

In the speed disciplines (super-G and downhill), poles are utilized primarily for initial acceleration and balance; while in the technical disciplines, the pole plant also helps to rotate the body while initiating a turn (Müller et al., 1998), as well as to clear the gates in the case of slalom. Accordingly, speed skiers use longer poles that are shaped around their body for better tuck and less aerodynamic drag (Barelle et al., 2004; Meyer et al., 2012).

### Racing Suit, Protective Devices, and Wearable Technologies

Small differences in aerodynamic drag can exert a major impact on skiing speed and properly fitted suits with low permeability provide less drag. Therefore, individualized suits for each discipline are designed with the average speed in mind (Brownlie et al., 2010; Bardal and Reid, 2012). In alpine skiing helmets are used primarily for safety, but at the same time, they contribute substantially to aerodynamic drag, particularly in the tuck position (Thompson et al., 2001). In addition to helmets that protect the athlete’s head from injury, the use of various other protective devices – i.e., protectors for the hand/arm, back, knee and lower-leg, knee orthoses and airbag systems – has been proposed in recent years (Spörri et al., 2017).

To optimize performance and/or ski equipment with respect to various biomechanical parameters (Supej et al., 2011; Hébert-Losier et al., 2014), skiers today are often equipped with wearable technologies, such as global navigation satellite systems (GNSS), inertial motion capture systems, accelerometers and sensors that measure GRF (Brodie et al., 2008; Krüger and Edelmann-Nusser, 2010; Supej, 2010; Vaverka and Vodickova, 2010; Supej and Holmberg, 2011; Nemec et al., 2014; Fasel et al., 2016; Falda-Buscaiot et al., 2017; Gilgien et al., 2018). Depending on the purpose and information required, different sensor technologies are used. When interference with the athlete must be minimized or there are other special needs/limitations, external devices such as photocells, radar guns and video recorders are employed (Krosshaug et al., 2007; Federolf et al., 2008; Supej and Holmberg, 2011; Supej et al., 2011, 2015; Federolf, 2012; Spörri et al., 2012b).

## Tactical Aspects of Olympic Alpine Skiing

When skiers have mastered techniques, racing tactics become important, varying with ability and external conditions. In all disciplines, gate combinations, the course setup, and snow conditions influence tactical considerations. At the same time, the athletes must gain as much time as possible on sections that emphasize their strengths and minimize loss of time on sections that expose their weaknesses (Hébert-Losier et al., 2014).

Overall, the key to success appears to be more closely related to a skier’s ability to maintain high-level performance, selecting the optimal turning technique and line of skiing, than achieving the fastest section time or highest instantaneous velocity (Hébert-Losier et al., 2014).

## Future Perspectives

The margins between the times that result in gold and silver medals in Olympic alpine skiing are hundredths of a second (e.g., this difference in the case of the women’s SG in Pyeongchang 2018 was 0.01 s), making all factors that influence performance extremely important. Although the biomechanics of alpine skiers have improved in recent decades, relatively little is yet known concerning optimization of performance over an entire course (Hébert-Losier et al., 2014) or interrelationships between skiing on successive sections (Supej and Cernigoj, 2006). Recent advances in GNSS technology allow precise biomechanical analysis of performance over an entire course in real-time (Supej et al., 2008, 2013; Supej, 2012; Gilgien et al., 2013), providing much more detailed information about such factors. In addition to measuring performance, inertial motion sensors and GNSS allow recording of 3D body kinematics over several turns or even an entire race course, providing accurate kinematic values on-snow (Brodie et al., 2008; Krüger and Edelmann-Nusser, 2010; Supej, 2010; Fasel et al., 2017). Continuous miniaturization of mechanical, electrical and optical sensing technologies for assessing the kinematics and kinetics of human motion and performance, as well as of other chemical sensing technologies designed to detect physiological parameters (not dealt with here) will allow more comfortable and flexible monitoring of technique, performance, tactics and training load (Heikenfeld et al., 2018). More user-friendly and automated software involving artificial intelligence (machine learning, neural networks and deep learning), in combination with wearable technology, is expected to allow real-time feedback in the near future (Nemec et al., 2014).

## Conclusion

In connection with future Olympic Games, regular and effective use of measurement technology and biomechanical feedback will improve and facilitate the work of coaches. Accordingly, both coaches and competitors will have to learn how to utilize novel technological possibilities for more efficient testing and selection of racing gear. The technical, physical and tactical strengths and weaknesses of an individual skier will become easier to identify. At the same time, the need to individualize training and skiing equipment will continue to motivate innovative scientific research for years to come.

## Author Contributions

MS and H-CH contributed substantially to all parts of this paper, including the concept, designed, and wrote, and approved the final version for publication, as well as agreed to be accountable for all aspects of this work.

## Conflict of Interest Statement

The authors declare that the research was conducted in the absence of any commercial or financial relationships that could be construed as a potential conflict of interest.

## Footnotes

1 www.fis-ski.com
